# (Cyclo­hexa­necarboxyl­ato)bis­(di-2-pyridylamine)zinc(II) nitrate monohydrate

**DOI:** 10.1107/S1600536809033443

**Published:** 2009-08-29

**Authors:** Ying-Jie Cai, Jun Yang, Peng Huang, Lei Lei, Qing-Fu Zeng

**Affiliations:** a Engineering Research Center for Clean Production of Textile Dyeing and Printing, Ministry of Education, Wuhan 430073, People’s Republic of China

## Abstract

In the title compound, [Zn(C_7_H_11_O_2_)(C_10_H_9_N_3_)_2_]NO_3_·H_2_O, the Zn^II^ atom is five-coordinated by two bidentate di-2-pyridylamine ligands and one O atom from a cyclo­hexa­necarboxy­ate anion, resulting in a ZnON_4_ square-based pyramidal coordination for the metal ion with the O atom in one of the basal positions. In the crystal, the components inter­act by way of O—H⋯O, O—H⋯N and N—H⋯O hydrogen bonds.

## Related literature

For background to acid and amine metal complexes and their mol­ecular architectures, see: Yang *et al.* (2004 ▶). For reference structural data, see: Allen *et al.* (1987 ▶).
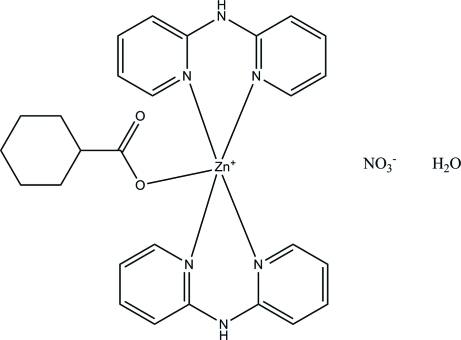


## Experimental

### 

#### Crystal data

[Zn(C_7_H_11_O_2_)(C_10_H_9_N_3_)_2_]NO_3_·H_2_O
*M* *_r_* = 614.96
Triclinic, 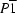

*a* = 10.4856 (3) Å
*b* = 11.6116 (13) Å
*c* = 13.4876 (13) Å
α = 107.526 (3)°
β = 106.016 (3)°
γ = 99.706 (3)°
*V* = 1447.3 (2) Å^3^
*Z* = 2
Mo *K*α radiation
μ = 0.90 mm^−1^
*T* = 296 K
0.32 × 0.26 × 0.20 mm

#### Data collection

Enraf–Nonius CAD-4 diffractometer
Absorption correction: ψ scan (North *et al.*, 1968 ▶) *T*_min_ = 0.761, *T*_max_ = 0.840
8944 measured reflections
6182 independent reflections
5178 reflections with *I* > 2σ(*I*)
*R*_int_ = 0.014
200 standard reflections every 3 reflections intensity decay: 1%

#### Refinement

*R*[*F* ^2^ > 2σ(*F* ^2^)] = 0.039
*wR*(*F* ^2^) = 0.109
*S* = 1.03
6182 reflections
386 parameters
3 restraints
H atoms treated by a mixture of independent and constrained refinement
Δρ_max_ = 0.46 e Å^−3^
Δρ_min_ = −0.34 e Å^−3^

### 

Data collection: *CAD-4 Software* (Enraf–Nonius, 1989 ▶); cell refinement: *CAD-4 Software*; data reduction: *XCAD4* (Harms & Wocadlo, 1995 ▶); program(s) used to solve structure: *SHELXS97* (Sheldrick, 2008 ▶); program(s) used to refine structure: *SHELXL97* (Sheldrick, 2008 ▶); molecular graphics: *SHELXTL* (Sheldrick, 2008 ▶); software used to prepare material for publication: *SHELXTL*.

## Supplementary Material

Crystal structure: contains datablocks global, I. DOI: 10.1107/S1600536809033443/hb5052sup1.cif

Structure factors: contains datablocks I. DOI: 10.1107/S1600536809033443/hb5052Isup2.hkl

Additional supplementary materials:  crystallographic information; 3D view; checkCIF report

## Figures and Tables

**Table 1:** Selected bond lengths (Å)

Zn1—N1	1.9984 (18)
Zn1—N3	2.0192 (19)
Zn1—N4	2.166 (2)
Zn1—N6	2.0405 (19)
Zn1—O2	1.9803 (16)

**Table 2:** Hydrogen-bond geometry (Å, °)

*D*—H⋯*A*	*D*—H	H⋯*A*	*D*⋯*A*	*D*—H⋯*A*
N12—H12⋯O6^i^	0.71 (3)	2.10 (3)	2.806 (3)	174 (3)
N11—H11*A*⋯O1^ii^	0.70 (2)	2.11 (3)	2.810 (3)	177 (3)
O6—H6*B*⋯N7^ii^	0.836 (10)	2.586 (14)	3.406 (4)	167 (3)
O6—H6*B*⋯O5^ii^	0.836 (10)	2.48 (3)	3.164 (4)	139 (3)
O6—H6*B*⋯O4^ii^	0.836 (10)	2.088 (12)	2.904 (4)	165 (3)
O6—H6*A*⋯O5^iii^	0.838 (10)	1.971 (15)	2.787 (4)	164 (4)

Symmetry codes: (i) 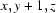; (ii) 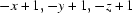; (iii) 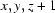.

**Fig. 1.**
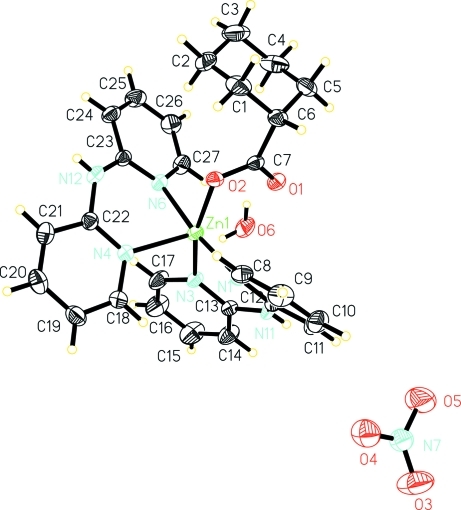
The molecular structure of (I) showing 30% probability displacement ellipsoids.

## supplementary crystallographic information

### Comment

There has been much research interest in the acid and amine metal complexes due to their molecular architectures (e.g. Yang *et al.*, 2004). In this work, we report here the crystal structure of the title compound, (I). In (I), all bond lengths are within normal ranges (Allen *et al.*, 1987) (Fig. 1). The Zn^II^ atom is five-coordinated by four N atoms from di-2-pyridylamine and one O atom from cyclohexanecarboxylic acid.

### Experimental

A mixture of di-2-pyridylamine (342 mg, 2 mmol), cyclohexanecarboxylic acid (256 mg, 2 mmol) and ZnNO_3_.6H_2_O (1 mmol, 297 mg) in methanol (10 ml) was stirred for 3 h. After keeping the filtrate in air for 8 d, colourless blocks of (I) were formed.

### Refinement

The N- and O-bound H atoms were located in a difference map and their positions were freely refined. The C-bound H atoms were positioned geometrically (C—H = 0.93 Å for the aromatic H atoms and C—H = 0.96 Å for the aliphatic H atoms) and were refined as riding, with *U*_iso_(H) = 1.2*U*_eq_(C).

### Crystal data

**Table:**

[Zn(C_7_H_11_O_2_)(C_10_H_9_N_3_)_2_]NO_3_·H_2_O	*Z* = 2
*M**_r_* = 614.96	*F*(000) = 640
Triclinic, *P*1	*D*_x_ = 1.411 Mg m^−^^3^
Hall symbol: -P 1	Mo *K*α radiation, λ = 0.71073 Å
*a* = 10.4856 (3) Å	Cell parameters from 25 reflections
*b* = 11.6116 (13) Å	θ = 9–12°
*c* = 13.4876 (13) Å	µ = 0.90 mm^−^^1^
α = 107.526 (3)°	*T* = 296 K
β = 106.016 (3)°	Block, colorless
γ = 99.706 (3)°	0.32 × 0.26 × 0.20 mm
*V* = 1447.3 (2) Å^3^	

### Data collection

**Table:**

Enraf–Nonius CAD-4 diffractometer	5178 reflections with *I* > 2σ(*I*)
Radiation source: fine-focus sealed tube	*R*_int_ = 0.014
graphite	θ_max_ = 27.0°, θ_min_ = 1.7°
ω/2θ scans	*h* = −12→13
Absorption correction: ψ scan (North *et al.*, 1968)	*k* = −14→13
*T*_min_ = 0.761, *T*_max_ = 0.840	*l* = −17→17
8944 measured reflections	200 standard reflections every 3 reflections
6182 independent reflections	intensity decay: 1%

### Refinement

**Table:**

Refinement on *F*^2^	Primary atom site location: structure-invariant direct methods
Least-squares matrix: full	Secondary atom site location: difference Fourier map
*R*[*F*^2^ > 2σ(*F*^2^)] = 0.039	Hydrogen site location: inferred from neighbouring sites
*wR*(*F*^2^) = 0.109	H atoms treated by a mixture of independent and constrained refinement
*S* = 1.03	*w* = 1/[σ^2^(*F*_o_^2^) + (0.0559*P*)^2^ + 0.6885*P*] where *P* = (*F*_o_^2^ + 2*F*_c_^2^)/3
6182 reflections	(Δ/σ)_max_ = 0.001
386 parameters	Δρ_max_ = 0.46 e Å^−^^3^
3 restraints	Δρ_min_ = −0.34 e Å^−^^3^

### Special details

**Table:**

Geometry. All e.s.d.'s (except the e.s.d. in the dihedral angle between two l.s. planes) are estimated using the full covariance matrix. The cell e.s.d.'s are taken into account individually in the estimation of e.s.d.'s in distances, angles and torsion angles; correlations between e.s.d.'s in cell parameters are only used when they are defined by crystal symmetry. An approximate (isotropic) treatment of cell e.s.d.'s is used for estimating e.s.d.'s involving l.s. planes.
Refinement. Refinement of *F*^2^ against ALL reflections. The weighted *R*-factor *wR* and goodness of fit *S* are based on *F*^2^, conventional *R*-factors *R* are based on *F*, with *F* set to zero for negative *F*^2^. The threshold expression of *F*^2^ > σ(*F*^2^) is used only for calculating *R*-factors(gt) *etc*. and is not relevant to the choice of reflections for refinement. *R*-factors based on *F*^2^ are statistically about twice as large as those based on *F*, and *R*- factors based on ALL data will be even larger.

### Fractional atomic coordinates and isotropic or equivalent isotropic displacement parameters (Å^2^)

**Table:**

	*x*	*y*	*z*	*U*_iso_*/*U*_eq_	
C1	0.1292 (3)	0.8840 (3)	0.4961 (3)	0.0615 (8)	
H1A	0.0511	0.8886	0.4402	0.074*	
H1B	0.2095	0.9486	0.5075	0.074*	
C2	0.1004 (4)	0.9075 (3)	0.6045 (3)	0.0724 (10)	
H2A	0.1822	0.9114	0.6623	0.087*	
H2B	0.0798	0.9878	0.6267	0.087*	
C3	−0.0192 (4)	0.8051 (4)	0.5925 (3)	0.0811 (11)	
H3A	−0.0298	0.8188	0.6640	0.097*	
H3B	−0.1035	0.8086	0.5422	0.097*	
C4	0.0032 (3)	0.6775 (4)	0.5488 (3)	0.0757 (10)	
H4A	0.0802	0.6703	0.6039	0.091*	
H4B	−0.0783	0.6135	0.5363	0.091*	
C5	0.0320 (3)	0.6546 (3)	0.4428 (3)	0.0630 (8)	
H5A	−0.0489	0.6527	0.3853	0.076*	
H5B	0.0505	0.5735	0.4198	0.076*	
C6	0.1545 (3)	0.7559 (3)	0.4557 (2)	0.0450 (6)	
H6	0.1607	0.7412	0.3819	0.054*	
C7	0.2903 (2)	0.7493 (2)	0.52994 (18)	0.0344 (5)	
C8	0.5875 (3)	0.8287 (3)	0.4789 (2)	0.0449 (6)	
H8	0.5734	0.9075	0.5050	0.054*	
C9	0.5912 (3)	0.7849 (3)	0.3745 (2)	0.0601 (8)	
H9	0.5806	0.8332	0.3304	0.072*	
C10	0.6110 (3)	0.6673 (3)	0.3351 (2)	0.0602 (8)	
H10	0.6114	0.6346	0.2632	0.072*	
C11	0.6299 (3)	0.5993 (3)	0.4021 (2)	0.0468 (6)	
H11	0.6429	0.5198	0.3767	0.056*	
C12	0.6293 (2)	0.6519 (2)	0.51007 (17)	0.0320 (4)	
C13	0.7019 (2)	0.6277 (2)	0.69238 (19)	0.0367 (5)	
C14	0.7804 (3)	0.5631 (3)	0.7447 (3)	0.0608 (8)	
H14	0.7979	0.4917	0.7031	0.073*	
C15	0.8314 (4)	0.6060 (4)	0.8583 (3)	0.0803 (11)	
H15	0.8851	0.5649	0.8946	0.096*	
C16	0.8021 (4)	0.7106 (4)	0.9178 (3)	0.0787 (11)	
H16	0.8352	0.7411	0.9949	0.094*	
C17	0.7240 (3)	0.7685 (3)	0.8619 (2)	0.0610 (8)	
H17	0.7060	0.8400	0.9029	0.073*	
C18	0.8081 (3)	1.0300 (3)	0.7532 (2)	0.0543 (7)	
H18	0.8350	0.9632	0.7146	0.065*	
C19	0.8960 (3)	1.1469 (3)	0.7940 (2)	0.0580 (7)	
H19	0.9805	1.1598	0.7835	0.070*	
C20	0.8562 (3)	1.2454 (3)	0.8512 (2)	0.0598 (8)	
H20	0.9138	1.3267	0.8801	0.072*	
C21	0.7314 (3)	1.2231 (2)	0.8654 (2)	0.0532 (7)	
H21	0.7030	1.2891	0.9036	0.064*	
C22	0.6474 (2)	1.1005 (2)	0.82200 (19)	0.0389 (5)	
C23	0.4434 (2)	0.9739 (2)	0.84199 (18)	0.0366 (5)	
C24	0.3436 (3)	0.9918 (3)	0.8921 (2)	0.0487 (6)	
H24	0.3325	1.0716	0.9202	0.058*	
C25	0.2633 (3)	0.8919 (3)	0.8994 (2)	0.0569 (7)	
H25	0.1964	0.9027	0.9317	0.068*	
C26	0.2822 (3)	0.7736 (3)	0.8580 (2)	0.0558 (7)	
H26	0.2298	0.7041	0.8634	0.067*	
C27	0.3796 (3)	0.7623 (3)	0.8094 (2)	0.0488 (6)	
H27	0.3922	0.6830	0.7817	0.059*	
H6A	0.524 (2)	0.360 (2)	0.983 (3)	0.081 (13)*	
H6B	0.386 (2)	0.322 (3)	0.921 (3)	0.087 (13)*	
N1	0.60361 (18)	0.76244 (17)	0.54675 (15)	0.0325 (4)	
N3	0.6709 (2)	0.72807 (19)	0.74978 (16)	0.0398 (4)	
N4	0.6839 (2)	1.00475 (19)	0.76529 (17)	0.0429 (5)	
N6	0.4594 (2)	0.85966 (18)	0.79898 (16)	0.0375 (4)	
N7	0.7893 (3)	0.5522 (2)	0.1219 (2)	0.0611 (6)	
O1	0.30686 (18)	0.64659 (15)	0.53325 (15)	0.0455 (4)	
O2	0.38469 (16)	0.85182 (15)	0.58651 (13)	0.0378 (4)	
O3	0.8755 (3)	0.5011 (3)	0.1033 (3)	0.1179 (12)	
O4	0.8103 (3)	0.6659 (2)	0.1560 (2)	0.0860 (8)	
O5	0.6740 (3)	0.4912 (3)	0.1109 (3)	0.1178 (12)	
O6	0.4564 (4)	0.2978 (2)	0.9418 (3)	0.0855 (8)	
N11	0.6563 (2)	0.58533 (19)	0.57844 (16)	0.0363 (4)	
N12	0.5229 (2)	1.0789 (2)	0.83921 (19)	0.0437 (5)	
Zn1	0.54528 (3)	0.81698 (2)	0.67867 (2)	0.03606 (10)	
H11A	0.667 (3)	0.528 (2)	0.553 (2)	0.027 (7)*	
H12	0.505 (3)	1.135 (3)	0.861 (2)	0.037 (8)*	

### Atomic displacement parameters (Å^2^)

**Table:**

	*U*^11^	*U*^22^	*U*^33^	*U*^12^	*U*^13^	*U*^23^
C1	0.0498 (16)	0.0601 (17)	0.083 (2)	0.0269 (14)	0.0102 (15)	0.0427 (16)
C2	0.066 (2)	0.067 (2)	0.073 (2)	0.0456 (17)	0.0105 (16)	0.0076 (16)
C3	0.056 (2)	0.121 (3)	0.085 (2)	0.048 (2)	0.0373 (18)	0.040 (2)
C4	0.0456 (17)	0.090 (2)	0.116 (3)	0.0254 (16)	0.0374 (19)	0.060 (2)
C5	0.0374 (14)	0.0568 (17)	0.075 (2)	0.0109 (13)	0.0036 (14)	0.0128 (15)
C6	0.0397 (13)	0.0597 (16)	0.0391 (13)	0.0209 (12)	0.0104 (10)	0.0218 (12)
C7	0.0345 (11)	0.0421 (12)	0.0335 (11)	0.0168 (10)	0.0169 (9)	0.0154 (10)
C8	0.0436 (13)	0.0501 (14)	0.0537 (15)	0.0196 (11)	0.0218 (12)	0.0279 (12)
C9	0.0623 (18)	0.086 (2)	0.0517 (16)	0.0263 (16)	0.0260 (14)	0.0439 (16)
C10	0.0665 (19)	0.084 (2)	0.0364 (14)	0.0240 (16)	0.0255 (13)	0.0226 (14)
C11	0.0501 (15)	0.0530 (15)	0.0358 (12)	0.0157 (12)	0.0206 (11)	0.0086 (11)
C12	0.0262 (10)	0.0358 (11)	0.0314 (11)	0.0059 (8)	0.0114 (9)	0.0088 (9)
C13	0.0381 (12)	0.0401 (12)	0.0372 (12)	0.0152 (10)	0.0171 (10)	0.0152 (10)
C14	0.076 (2)	0.0703 (19)	0.0535 (16)	0.0487 (17)	0.0255 (15)	0.0284 (15)
C15	0.097 (3)	0.107 (3)	0.0597 (19)	0.068 (2)	0.0225 (19)	0.045 (2)
C16	0.090 (3)	0.118 (3)	0.0367 (15)	0.062 (2)	0.0150 (16)	0.0287 (17)
C17	0.0690 (19)	0.083 (2)	0.0332 (13)	0.0451 (17)	0.0160 (13)	0.0129 (13)
C18	0.0391 (14)	0.0557 (16)	0.0552 (16)	0.0088 (12)	0.0171 (12)	0.0042 (13)
C19	0.0411 (14)	0.0645 (18)	0.0552 (16)	−0.0017 (13)	0.0155 (13)	0.0138 (14)
C20	0.0596 (18)	0.0477 (16)	0.0573 (17)	−0.0043 (13)	0.0121 (14)	0.0167 (14)
C21	0.0624 (17)	0.0370 (13)	0.0538 (16)	0.0108 (12)	0.0182 (14)	0.0115 (12)
C22	0.0408 (12)	0.0394 (12)	0.0345 (12)	0.0118 (10)	0.0108 (10)	0.0124 (10)
C23	0.0392 (12)	0.0414 (12)	0.0290 (11)	0.0167 (10)	0.0111 (9)	0.0101 (9)
C24	0.0515 (15)	0.0582 (16)	0.0423 (13)	0.0253 (13)	0.0236 (12)	0.0141 (12)
C25	0.0533 (16)	0.078 (2)	0.0512 (16)	0.0224 (15)	0.0314 (14)	0.0246 (15)
C26	0.0579 (17)	0.0620 (17)	0.0546 (16)	0.0104 (14)	0.0288 (14)	0.0260 (14)
C27	0.0596 (16)	0.0433 (14)	0.0465 (14)	0.0137 (12)	0.0241 (13)	0.0157 (11)
N1	0.0304 (9)	0.0378 (10)	0.0343 (9)	0.0121 (8)	0.0149 (8)	0.0155 (8)
N3	0.0423 (11)	0.0490 (11)	0.0309 (9)	0.0230 (9)	0.0135 (8)	0.0121 (9)
N4	0.0361 (10)	0.0399 (11)	0.0455 (11)	0.0080 (8)	0.0160 (9)	0.0054 (9)
N6	0.0414 (11)	0.0384 (10)	0.0350 (10)	0.0139 (8)	0.0172 (8)	0.0113 (8)
N7	0.0612 (16)	0.0498 (15)	0.0746 (17)	0.0120 (12)	0.0294 (14)	0.0227 (13)
O1	0.0478 (10)	0.0371 (9)	0.0546 (10)	0.0202 (8)	0.0181 (8)	0.0160 (8)
O2	0.0335 (8)	0.0392 (9)	0.0431 (9)	0.0148 (7)	0.0138 (7)	0.0152 (7)
O3	0.101 (2)	0.087 (2)	0.199 (4)	0.0436 (18)	0.093 (3)	0.051 (2)
O4	0.0831 (18)	0.0619 (15)	0.116 (2)	0.0114 (13)	0.0389 (16)	0.0373 (14)
O5	0.0797 (19)	0.0729 (18)	0.176 (3)	−0.0024 (15)	0.059 (2)	0.0110 (19)
O6	0.094 (2)	0.0605 (15)	0.097 (2)	0.0444 (16)	0.0289 (17)	0.0132 (14)
N11	0.0434 (11)	0.0309 (10)	0.0356 (10)	0.0146 (9)	0.0171 (9)	0.0081 (9)
N12	0.0512 (13)	0.0375 (11)	0.0473 (12)	0.0212 (10)	0.0235 (10)	0.0116 (10)
Zn1	0.03508 (15)	0.03867 (16)	0.03545 (15)	0.01457 (11)	0.01437 (11)	0.01066 (11)

### Geometric parameters (Å, °)

**Table:**

C1—C6	1.518 (4)	C16—C17	1.356 (4)
C1—C2	1.529 (5)	C16—H16	0.9300
C1—H1A	0.9700	C17—N3	1.356 (3)
C1—H1B	0.9700	C17—H17	0.9300
C2—C3	1.511 (5)	C18—N4	1.351 (3)
C2—H2A	0.9700	C18—C19	1.357 (4)
C2—H2B	0.9700	C18—H18	0.9300
C3—C4	1.506 (5)	C19—C20	1.372 (4)
C3—H3A	0.9700	C19—H19	0.9300
C3—H3B	0.9700	C20—C21	1.367 (4)
C4—C5	1.497 (5)	C20—H20	0.9300
C4—H4A	0.9700	C21—C22	1.392 (4)
C4—H4B	0.9700	C21—H21	0.9300
C5—C6	1.519 (4)	C22—N4	1.326 (3)
C5—H5A	0.9700	C22—N12	1.383 (3)
C5—H5B	0.9700	C23—N6	1.337 (3)
C6—C7	1.527 (3)	C23—N12	1.373 (3)
C6—H6	0.9800	C23—C24	1.406 (3)
C7—O1	1.246 (3)	C24—C25	1.360 (4)
C7—O2	1.272 (3)	C24—H24	0.9300
C8—N1	1.356 (3)	C25—C26	1.389 (4)
C8—C9	1.360 (4)	C25—H25	0.9300
C8—H8	0.9300	C26—C27	1.361 (4)
C9—C10	1.382 (4)	C26—H26	0.9300
C9—H9	0.9300	C27—N6	1.357 (3)
C10—C11	1.365 (4)	C27—H27	0.9300
C10—H10	0.9300	N7—O3	1.204 (4)
C11—C12	1.402 (3)	N7—O4	1.218 (3)
C11—H11	0.9300	N7—O5	1.239 (4)
C12—N1	1.332 (3)	Zn1—N1	1.9984 (18)
C12—N11	1.373 (3)	Zn1—N3	2.0192 (19)
C13—N3	1.337 (3)	Zn1—N4	2.166 (2)
C13—N11	1.376 (3)	Zn1—N6	2.0405 (19)
C13—C14	1.395 (3)	Zn1—O2	1.9803 (16)
C14—C15	1.370 (4)	O6—H6A	0.838 (10)
C14—H14	0.9300	O6—H6B	0.836 (10)
C15—C16	1.374 (5)	N11—H11A	0.70 (2)
C15—H15	0.9300	N12—H12	0.71 (3)
			
C6—C1—C2	110.8 (2)	N3—C17—C16	123.7 (3)
C6—C1—H1A	109.5	N3—C17—H17	118.2
C2—C1—H1A	109.5	C16—C17—H17	118.2
C6—C1—H1B	109.5	N4—C18—C19	124.1 (3)
C2—C1—H1B	109.5	N4—C18—H18	118.0
H1A—C1—H1B	108.1	C19—C18—H18	118.0
C3—C2—C1	111.4 (3)	C18—C19—C20	117.9 (3)
C3—C2—H2A	109.3	C18—C19—H19	121.1
C1—C2—H2A	109.3	C20—C19—H19	121.1
C3—C2—H2B	109.3	C21—C20—C19	119.6 (3)
C1—C2—H2B	109.3	C21—C20—H20	120.2
H2A—C2—H2B	108.0	C19—C20—H20	120.2
C4—C3—C2	111.1 (2)	C20—C21—C22	119.2 (3)
C4—C3—H3A	109.4	C20—C21—H21	120.4
C2—C3—H3A	109.4	C22—C21—H21	120.4
C4—C3—H3B	109.4	N4—C22—N12	119.7 (2)
C2—C3—H3B	109.4	N4—C22—C21	121.8 (2)
H3A—C3—H3B	108.0	N12—C22—C21	118.6 (2)
C5—C4—C3	112.1 (3)	N6—C23—N12	121.9 (2)
C5—C4—H4A	109.2	N6—C23—C24	121.3 (2)
C3—C4—H4A	109.2	N12—C23—C24	116.8 (2)
C5—C4—H4B	109.2	C25—C24—C23	119.6 (2)
C3—C4—H4B	109.2	C25—C24—H24	120.2
H4A—C4—H4B	107.9	C23—C24—H24	120.2
C4—C5—C6	111.6 (3)	C24—C25—C26	119.4 (2)
C4—C5—H5A	109.3	C24—C25—H25	120.3
C6—C5—H5A	109.3	C26—C25—H25	120.3
C4—C5—H5B	109.3	C27—C26—C25	118.0 (3)
C6—C5—H5B	109.3	C27—C26—H26	121.0
H5A—C5—H5B	108.0	C25—C26—H26	121.0
C1—C6—C5	109.8 (2)	N6—C27—C26	123.9 (3)
C1—C6—C7	112.4 (2)	N6—C27—H27	118.0
C5—C6—C7	112.2 (2)	C26—C27—H27	118.0
C1—C6—H6	107.4	C12—N1—C8	117.9 (2)
C5—C6—H6	107.4	C12—N1—Zn1	123.08 (14)
C7—C6—H6	107.4	C8—N1—Zn1	117.45 (16)
O1—C7—O2	122.0 (2)	C13—N3—C17	117.2 (2)
O1—C7—C6	120.5 (2)	C13—N3—Zn1	123.65 (15)
O2—C7—C6	117.4 (2)	C17—N3—Zn1	119.08 (17)
N1—C8—C9	122.9 (3)	C22—N4—C18	117.5 (2)
N1—C8—H8	118.5	C22—N4—Zn1	122.70 (16)
C9—C8—H8	118.5	C18—N4—Zn1	119.52 (17)
C8—C9—C10	118.7 (3)	C23—N6—C27	117.6 (2)
C8—C9—H9	120.6	C23—N6—Zn1	122.08 (15)
C10—C9—H9	120.6	C27—N6—Zn1	117.11 (16)
C11—C10—C9	119.7 (2)	O3—N7—O4	122.9 (3)
C11—C10—H10	120.1	O3—N7—O5	121.4 (3)
C9—C10—H10	120.1	O4—N7—O5	115.7 (3)
C10—C11—C12	118.5 (3)	C7—O2—Zn1	108.91 (14)
C10—C11—H11	120.7	H6A—O6—H6B	109.5 (17)
C12—C11—H11	120.7	C12—N11—C13	129.1 (2)
N1—C12—N11	120.21 (19)	C12—N11—H11A	113 (2)
N1—C12—C11	122.0 (2)	C13—N11—H11A	115 (2)
N11—C12—C11	117.8 (2)	C23—N12—C22	131.3 (2)
N3—C13—N11	120.1 (2)	C23—N12—H12	114 (2)
N3—C13—C14	121.9 (2)	C22—N12—H12	113 (2)
N11—C13—C14	118.0 (2)	O2—Zn1—N1	89.03 (7)
C15—C14—C13	119.2 (3)	O2—Zn1—N3	162.61 (8)
C15—C14—H14	120.4	N1—Zn1—N3	88.38 (7)
C13—C14—H14	120.4	O2—Zn1—N6	85.24 (7)
C14—C15—C16	119.1 (3)	N1—Zn1—N6	172.28 (8)
C14—C15—H15	120.4	N3—Zn1—N6	95.55 (8)
C16—C15—H15	120.4	O2—Zn1—N4	100.52 (8)
C17—C16—C15	118.8 (3)	N1—Zn1—N4	99.74 (8)
C17—C16—H16	120.6	N3—Zn1—N4	96.86 (9)
C15—C16—H16	120.6	N6—Zn1—N4	86.42 (8)

### Hydrogen-bond geometry (Å, °)

**Table:**

*D*—H···*A*	*D*—H	H···*A*	*D*···*A*	*D*—H···*A*
N12—H12···O6^i^	0.71 (3)	2.10 (3)	2.806 (3)	174 (3)
N11—H11A···O1^ii^	0.70 (2)	2.11 (3)	2.810 (3)	177 (3)
O6—H6B···N7^ii^	0.84 (1)	2.59 (1)	3.406 (4)	167 (3)
O6—H6B···O5^ii^	0.84 (1)	2.48 (3)	3.164 (4)	139 (3)
O6—H6B···O4^ii^	0.84 (1)	2.09 (1)	2.904 (4)	165 (3)
O6—H6A···O5^iii^	0.84 (1)	1.97 (2)	2.787 (4)	164 (4)

Symmetry codes: (i) *x*, *y*+1, *z*; (ii) −*x*+1, −*y*+1, −*z*+1; (iii) *x*, *y*, *z*+1.
